# Supplementary material for: A Selective ALDH1A3 Inhibitor Impairs Mesothelioma 3-D Multicellular Spheroid Growth and Neutrophil Recruitment
Source: Int J Mol Sci. 2023 Apr 3;24(7):6689. doi: 10.3390/ijms24076689 (PMC10094992; doi:10.3390/ijms24076689)

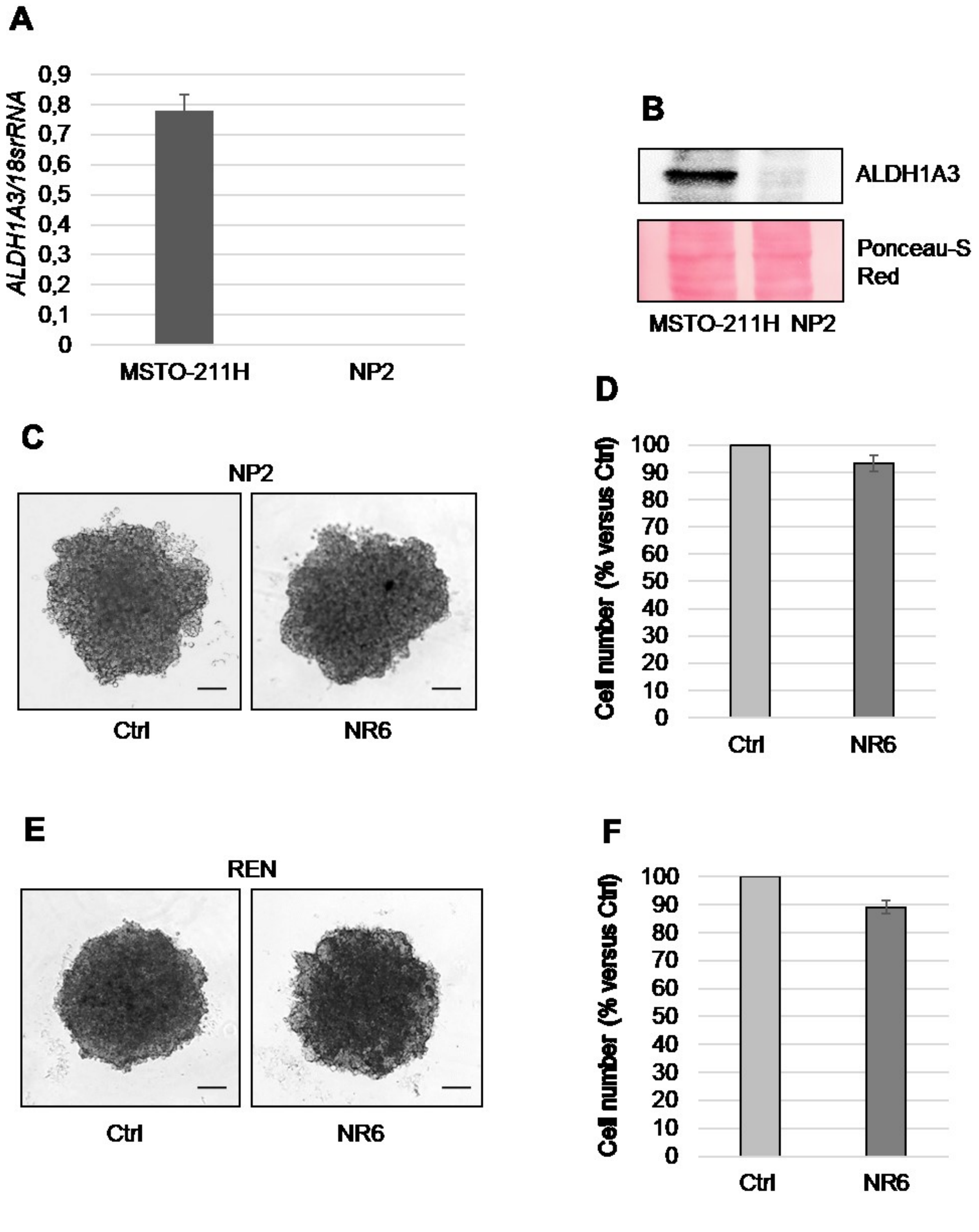

Figure S1. Response of ALDH1A3 negative MPM REN and mesothelial NP2 cells to NR6 treatment.(A) Bar graph shows *ALDH1A3* mRNA expression evaluated by real time-PCR in MSTO-211H and NP2 MCSs, at 72 hours. Data are expressed as *ALDH1A3* mRNA/*18S* rRNA ratio. (B) Representative Western Blot analysis of ALDH1A3 expression in MSTO-211H and NP2 MCSs, at 72 hours. Whole cell proteins were visualized by Ponceau-S Red staining. (C) Representative phase contrast images (x40 magnification) of NP2 cells cultured as MCSs +/- NR6 treatment, for 72 hours. Scale bar = 100 µm. (D) Bar graph shows the number of viable cells in NP2 MCSs treated, 72 hours, with NR6 represented as percentage versus untreated control MCSs (Ctrl). (E) Representative phase contrast images (x40 magnification) of REN cells cultured as MCSs +/- NR6 treatment, for 72 hours. Scale bar = 100 µm. (F) Bar graph shows the number of viable cells in REN MCSs treated, 72 hours, with NR6 represented as percentage versus untreated control MCSs (Ctrl). In all graphs reported in Figure S1, each bar represents mean ± s.d. of three independent experiments.

LC-HRMS analysis

Instrumentation:	Hybrid quadrupole-orbitrap, Thermo Scientific Q-exactive *Plus*, equipped with a Vanquish UHPLC system.
Column:	Kinetex C18 (150 × 2.1 mm, 2.6 µm d_p) + C18 RP-Security Guard™ (Phenomenex).
Mobile Phase	Phase A: 0.1% formic acid in water UHPLC grade.<br>Phase B: 0.1% formic acid in methanol UHPLC grade.
Analysis mode:	Gradient of concentration.

Time (min)	% B
0,00	20
8,00	90
11,50	90
12,00	20
17,00	20

Flow rate:	0.250 mL/min
Column temperature:	40 °C
Sample temperature:	15 °C
Injected volume:	10 µL

The operating conditions of the HESI were as follows:

Parameter	Value
sheath gas flow rate (N_2)	45 Auxiliary Units
auxiliary gas flow rate	10 Auxiliary Units
sweep gas flow rate	0 Auxiliary Units
spray voltage	3.50 kV
capillary temperature	300 °C
auxiliary gas heater temperature	280 °C

Data were acquired in positive full scan e parallel reaction monitoring (PRM) modes:

Full scan	Value
Microscan	1
Resolution	70,000
AGC target	3e6

PRM (MS^2)	Value
Microscan	1
Resolution	35,000
AGC target	1e5
Maximum IT	120 ms
Loop count	1
MSX count	1
Isolation wind.	2.0 *m/z*
Isolation offset	0.5 *m/z*
NCE	30, 60, 90

LC-HRMS chromatogram and MS2 spectrum of MDA derivatized with DCHD

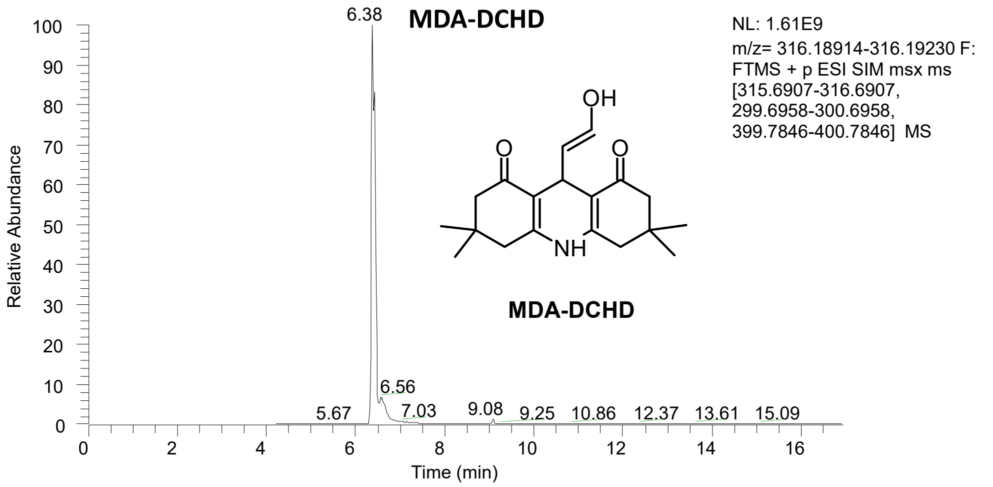

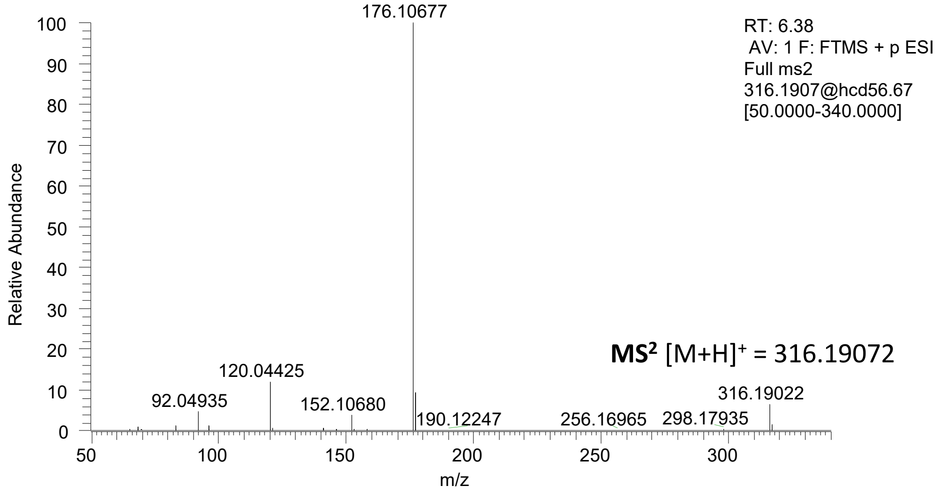

Supplement: Supplementary file 1 [file ijms-24-06689-s001.zip › ijms-2317294-supplementary.pdf]
